# Effects of Nanoparticle Size and Radiation Energy on Copper-Cysteamine Nanoparticles for X-ray Induced Photodynamic Therapy

**DOI:** 10.3390/nano10061087

**Published:** 2020-06-01

**Authors:** Bindeshwar Sah, Jing Wu, Adam Vanasse, Nil Kanatha Pandey, Lalit Chudal, Zhenzhen Huang, Wenzhi Song, Hongmei Yu, Lun Ma, Wei Chen, Michael P. Antosh

**Affiliations:** 1 Department of Physics, University of Rhode Island, 2 Lippitt Road, Kingston, RI 02881, USA; bindeshwar_sah@uri.edu (B.S.); adam_vanasse@uri.edu (A.V.); 2 Department of Computer Science and Statistics, University of Rhode Island, 9 Greenhouse Road, Kingston, RI 02881, USA; jing_wu@uri.edu; 3 Department of Physics, The University of Texas at Arlington, Arlington, TX 76019, USA; nilkanatha.pandey@mavs.uta.edu (N.K.P.); lalit.chudal@mavs.uta.edu (L.C.); lunma@mavs.uta.edu (L.M.); 4 College of Chemistry and Department of Stomatology, Jilin University, Changchun 130012, China; huangzhen@jlu.edu.cn (Z.H.); songwz@jlu.edu.cn (W.S.); 5 School of Chemical Engineering, University of Science and Technology Liaoning, Anshan 114051, China; yuhongmei@ustl.edu.cn; 6 Institute for Brain and Neural Systems, Brown University, 184 Hope Street, Providence, RI 02912, USA

**Keywords:** copper-cysteamine nanoparticle, mice, radiation energies, size, reactive oxygen species, tumor, X-ray, photodynamic therapy

## Abstract

The Copper-cysteamine (Cu-Cy) nanoparticle is a novel sensitizer with a potential to increase the effectiveness of radiation therapy for cancer treatment. In this work, the effect of nanoparticle size and the energy of X-rays on the effectiveness of radiation therapy are investigated. The effect of the particle size on their performance is very complicated. The nanoparticles with an average size of 300 nm have the most intense photoluminescence, the nanoparticles with the average size of 100 nm have the most reactive oxygen species production upon X-ray irradiation, while the nanoparticles with the average size of 40 nm have the best outcome in the tumor suppression in mice upon X-ray irradiation. For energy, 90 kVp radiation resulted in smaller tumor sizes than 250 kVp or 350 kVp radiation energies. Overall, knowledge of the effect of nanoparticle size and radiation energy on radiation therapy outcomes could be useful for future applications of Cu-Cy nanoparticles.

## 1. Introduction

Photodynamic therapy (PDT) has emerged as an efficient modality for cancer treatment with many advantages, including activated acute immune responses, negligible side effects, little intrinsic or acquired resistance, minimal invasiveness, and drug resistance [1,2,3,4]. There are three necessary components involved in the PDT process: a photosensitizer, light, and oxygen [1,5]. Light sources used in conventional PDT cannot penetrate deep tissues, thereby limiting its clinical application [6]. To solve this problem, several possible solutions have been proposed, such as inventing novel photosensitizers and developing up-conversion nanoparticles [7,8,9]. Recently, X-ray-induced photodynamic therapy (X-PDT) has attracted considerable attention owing to its limitless tissue penetration capacity [10,11,12,13,14].

Copper-cysteamine (Cu-Cy) nanoparticle [15] is a novel sensitizer that can be activated by various excitation sources such as ultraviolet (UV) light [15,16], microwave (MW) [17,18], X-ray [7,15,19,20], and ultrasound (US) [21] as well as a cancer-specific intracellular stimulating agent (H_2_O_2_/acidic pH) [22] to produce reactive oxygen species (ROS) for cancer treatment. Furthermore, the combination of Cu-Cy and potassium iodide (KI) was able to destroy both Gram-positive and Gram-negative bacteria when excited by UV light [23]. This evidence directs us to believe that Cu-Cy nanoparticles have the potential to be a next-generation nanomedicine for cancer treatment and bacterial inactivation. In particular, the ability of Cu-Cy nanoparticles to be activated by X-rays has received a great deal of attention among researchers as this would help to enhance existing radiotherapy by decreasing the radiation dose required to achieve the same therapeutic outcome, thereby reducing the occurrence of side-effects.

Since the initial discovery of Cu-Cy nanoparticles [15], significant work has been done to understand the properties of the nanoparticles. Recently, a proof of concept has been undertaken to demonstrate that Cu-Cy nanoparticles can be used to significantly reduce tumor size in vivo when combined with radiation therapy, compared to radiation therapy alone [20].

After a successful proof of concept, one logical step is to test the effect of clinically important variables. In this work, two variables are tested: nanoparticle size and radiation energy. Nanoparticle size can affect the probability of radiation interactions (for example, photon interactions are an exponential function of material thickness [24]), and could also impact the ability of the Cu-Cy nanoparticles to produce ROS (similar to how Auger electrons produced in gold nanoparticles sometimes have energies that are too low to escape from larger gold nanoparticles [25]). Nanoparticle uptake may be a non-linear function of size—for example, with gold nanoparticles, the literature suggests that smaller nanoparticles may go through tumors quickly, while larger nanoparticles may not be able to enter tumors. However, middle-sized nanoparticles are found to be ideal for radiation therapy [26,27,28,29]. Furthermore, radiation energy also affects the probability of radiation interactions, with the most common result being that higher radiation energies have lower interaction probabilities but higher radiation energy deposits per interaction on average [24]. Most radiation therapy done in clinics is with megavoltage sources, which are significantly higher than the radiation energies used in previous in vivo work [7,20].

Since radiation penetrates more deeply at higher energies, the range of tumors that can be effectively treated with radiation and Cu-Cy nanoparticles depends directly on the response to different radiation energies. Likewise, different sized nanoparticles may have different bio-distributions, and only a certain range of sizes may allow effective discharge of nanoparticles from the system. Knowing the effect of these important variables significantly improves any evaluation of the potential for Cu-Cy nanoparticles to effectively enhance radiation therapy in future clinical applications. In this contribution, for the first time, we have investigated the effects of different sizes of Cu-Cy nanoparticles and radiation energies on X-ray mediated photodynamic therapy.

## 2. Materials and Methods

### 2.1. Materials

Copper chloride dihydrate, cysteamine hydrochloride, sodium hydroxide (NaOH), p-nitrosodimethylaniline (RNO), and imidazole (ID) were bought from Sigma-Aldrich, USA.

### 2.2. Copper-Cysteamine (Cu-Cy) Nanoparticle Synthesis and Conjugation with pH-Low Insertion Peptide

Cu-Cy nanoparticles were synthesized in the laboratory of Wei Chen, following the method previously established [15]. Briefly, 91 mg of copper chloride dihydrate and 127 mg of cysteamine hydrochloride were dissolved into 25 mL of deionized (DI) water. After that, the pH value was adjusted to 8 by adding NaOH solution. The solution was then stirred at 750 rpm for about 2 h at room temperature. Then, the solution was heated for 30 min at the boiling point of water. Afterward, the solution was allowed to cool naturally at room temperature. As-prepared Cu-Cy particles were subsequently washed with the mixture of water and ethanol three times and dried in a vacuum oven overnight at 40 °C.

The as-prepared product was then dispersed in DI water using a bath sonicator for 1 h, and different sized Cu-Cy were separated by centrifugation. Briefly, the dispersed Cu-Cy particles were centrifuged at 1500 rpm for 1 min, and residue was removed. The as-formed supernatant was subsequently centrifuged at 1500 rpm for 1 min. The precipitate was removed again, and the supernatant was collected. Next, the supernatant was centrifuged at 3000 rpm for 2 min, and sediment was collected, called large-sized nanoparticles. In order to obtain medium-sized nanoparticles, the supernatant of large-sized nanoparticles was further centrifuged at 3000 rpm for 1 min, and residue was discarded. Afterward, the supernatant was centrifuged at 3000 rpm for 8 min, and residue was collected, labeled as medium-sized nanoparticles. To obtain small-sized nanoparticles, the supernatant of medium-sized nanoparticles was further centrifuged at 3000 rpm for 5 min, and residue was removed. After collecting the suspension, the suspension was further centrifuged at 6000 rpm for 20 min, and the residue was collected, called small-sized nanoparticles. pH-low insertion peptide (pHLIP) [30], a tumor-targeting molecule that targets tumors based on pH, was conjugated to the different sized Cu-Cy nanoparticles in order to aid with tumor uptake, using the same methodology as Shrestha et al. [20].

### 2.3. Nanoparticle Characterization

The size of the different sized Cu-Cy nanoparticles was studied by using a TEM-2100 HR transmission electron microscope (TEM, JEOL Ltd., Tokyo Japan). In order to compare the excitation and emission spectra of three different sized Cu-Cy nanoparticles, an equal amount of three different sized Cu-Cy nanoparticles were dispersed in DI water, and the photoluminescence (PL) spectra were measured using a Shimadzu RF-5301PC spectrofluorophotometer (Kyoto, Japan). The emission spectra of the samples were recorded by exciting the samples at 365 nm, whereas the excitation spectra were obtained using an emission wavelength of 607 nm under the identical experimental conditions.

### 2.4. Reactive Oxygen Species (ROS) Detection

The ROS generated by different sized Cu-Cy nanoparticles upon X-ray irradiation were measured using the *p*-nitrosodimethylaniline (RNO) and imidazole (ID) method [31]. Typically, 0.225 mg of RNO and 16.34 mg of ID were separately dissolved in 30 mL of DI water. These solutions were air saturated by sufficient air bubbling just before the experiment. The sample solution was prepared by taking 1 mL of RNO, 1 mL of ID, and 1 mL of Cu-Cy nanoparticles. Then, the absorption of the solution was monitored using a Shimadzu UV-2450 spectrophotometer (Kyoto, Japan at 440 nm after each irradiation (90 kVp) in the interval of 1 min. The control experiment was carried out following the same procedure except DI water was used instead of Cu-Cy nanoparticles.

### 2.5. Tumor Size Experiment

**Figure 1:**
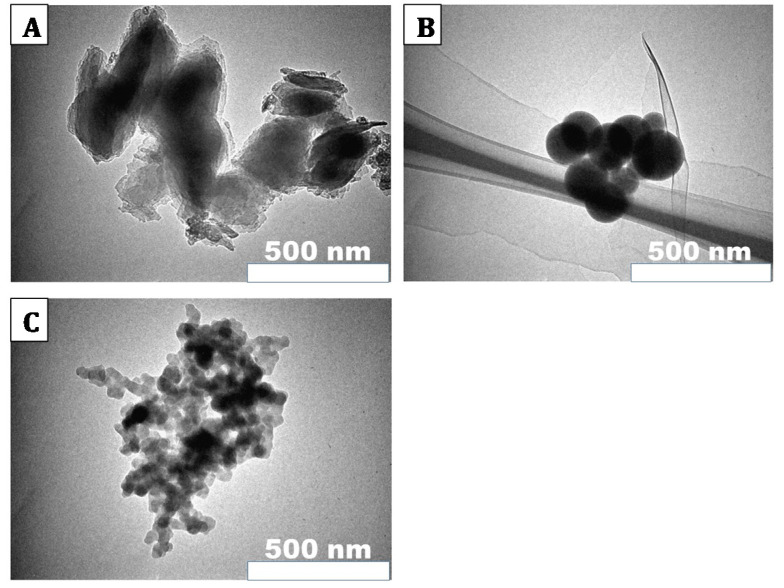
Representative transmission electron microscope (TEM) images of the three different-sized Cu-Cy nanoparticles: Large nanoparticles with an average size of about 300 nm (**A**), medium-sized nanoparticles with an average size of about 100 nm (**B**), and small-sized nanoparticles with an average size of about 40 nm (**C**).

This experiment was undertaken under protocol AN1516-003, approved by the Institutional Animal Care and Use Committee at the University of Rhode Island. 60 Balb/C mice, purchased from Envigo, were used in this experiment. (More mice were ordered, but this was the number of mice that grew tumors.) Mice were anesthetized (isoflurane gas anesthesia) each injected with approximately 1.5 million JC murine adenocarcinoma cells (purchased from American Type Culture Collection (ATCC), Manassas, USA) in cell medium (Roswell Park Memorial Institute). When tumors reached an approximate length of 5–8 mm, similar to [20,32,33], mice were anesthetized and injected intratumorally with a 20 μL solution containing 16 μg of Cu-Cy nanoparticles (conjugated to pHLIP) in phosphate buffer solution. Each mouse was given one of three different sizes of nanoparticles: mean size 40, 100, and 300 nm (see Figure 1A–C below).

Thirty minutes after injection of nanoparticles (similar to [20]) mice were anesthetized and irradiated in a cabinet X-ray machine (Faxitron MultiRad 350, IL, USA). A 5 gray dose of radiation was given to each mouse, as measured by a radical ion chamber dosimeter. For mice irradiated with 90 kVp energy, the maximum voltage was 90 kV and only internal tube filtering was used (similar to [7,20]); for 250 kVp and 350 kVp energy the maximum voltage was set to 250 and 350 kV, respectively and a Thoraeus-1 filter was used.

Mouse tumors were measured for one month after irradiation. Mice were euthanized at 28 days after irradiation, if maximum tumor length exceeded approximately 20 mm, or if skin became necrotic. Some (but not all) mice were tracked for 3–4 days past day 28; these extra measurements were ignored in the analysis and were not plotted. Supplementary Table S1 contains the treatment groups and the reason for euthanasia for each mouse in the experiment.

The experimental groups were as follows:

(Smallest nanoparticles + 90 kVp) (Note: this is the same group as in Shrestha et al. [20])
(Smallest nanoparticles + 250 kVp)
(Smallest nanoparticles + 350 kVp)
(Middle nanoparticles + 90 kVp)
(Largest nanoparticles + 90 kVp)

The number of mice in each group can be found specifically in the Supplementary Table S1.

### 2.6. Tumor Size Analysis

Tumor volume was calculated using the formula: volume = (1/2) * (length) * (width)^2^, where length is longer than width [34]. The longitudinal tumor size measurements of 60 mice between day 0 to 28 were considered in the analysis. Some mice were euthanized during the study when tumors reached a large size (approx. 20 mm length) or when the skin became necrotic. The reasons for dropout were thus known (listed in the Supplementary Table S1) and were also included in the analysis as covariates, which makes the missing data mechanism most likely missing at random (MAR) [35]. Given that the longitudinal data were MAR with dropout only, an observation-specific weighted generalized estimating equation (WGEE) [36] was performed on the tumor size data, where each measure was weighted by the inverse probability of being observed to reduce the bias caused by missing data. Covariates that potentially affecting the tumor size include day number after irradiation, sex (male or female), age of mouse at time after, tumor volume at time of irradiation, radiation energy, and nanoparticle size. All the continuous covariates were standardized. The identity link function was assumed and the compound symmetric working correlation structure was selected using the deviance information criterion (DIC) [37] based on completely observed data. A logistic regression model was fit for missingness, where 1 indicates observed and 0 indicates missing. Comparisons of treatment groups were done, including a calculation of statistical significance (*p*-value) for the difference between each of the three radiation energies (90 kVp, 250 kVp, and 350 kVp) and the three nanoparticle sizes (40, 100, and 300 nm), with corrections for multiple hypothesis testing using a Tukey–Kramer adjustment. 

## 3. Results

### 3.1. Study of Size Distribution, Photoluminescence (PL) Spectra, and ROS Production

**Figure 2:**
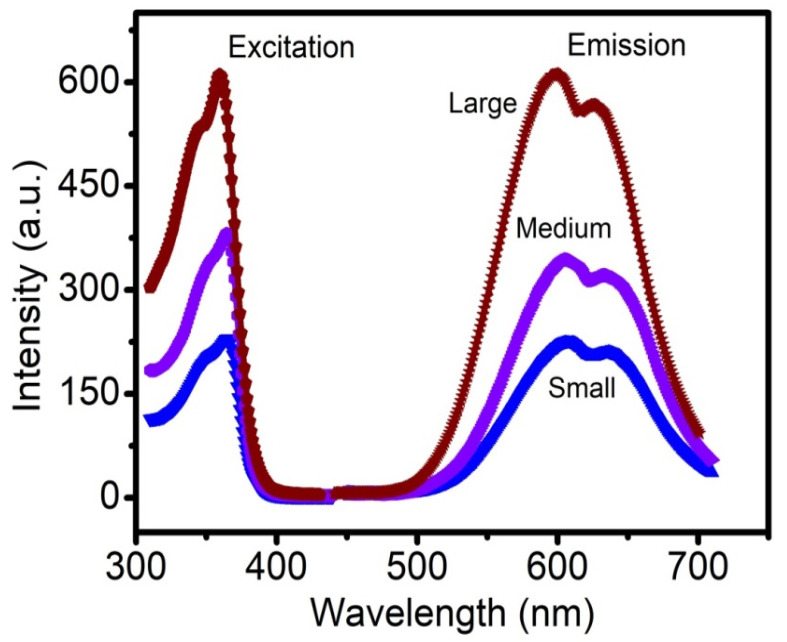
The photoluminescence (PL) excitation spectra (left) and emission spectra (right) of the large, medium, and small-sized Cu-Cy nanoparticles with excitation and emission wavelengths of 365 nm and 607 nm, respectively.

The size distribution of different-sized Cu-Cy nanoparticles was recorded using a transmission electron microscope (TEM) and representative photographs are shown in Figure 1. The average sizes of the three different-sized nanoparticles were 40 nm (small), 100 nm (medium), and 300 nm (large). The PL spectra of the different-sized Cu-Cy nanoparticles were recorded using the spectrofluorophotometer. Our results demonstrate that the PL intensity increased with the size of Cu-Cy nanoparticles (Figure 2), suggesting a vital role of the size in the PL intensity. As displayed in Figure 2, the PL intensity of the small-sized Cu-Cy nanoparticles was the lowest. Even though the exact mechanism of the increase in the PL intensity of Cu-Cy nanoparticles with the increase in size is not completely understood yet, there could be different possibilities as discussed below. The PL intensity of phosphors is strongly related to the physical properties of the materials, such as surface area, surface property, phase purity, and crystallinity [38]. Jung et al. reported that the particles (Y_2_O_3_:Eu) with a smaller surface area had a higher PL intensity. Besides that, they found a linear relationship between crystallite size and the PL intensity [38]. Furthermore, in another study by Wang et al., the authors reported the increase in the PL intensity of Y_2_O_3_:Eu^3+^ submicrometer particles while increasing the particle size and crystallite size [39]. Chen et al. found that both the size and the surface/volume ratio are key parameters to determine the luminescence efficiency of nanoparticles [40,41]. Based on these references, we would think that the reduction in the surface area (i.e., increase in nanoparticle size) could be one of the most likely mechanisms of the increase in the PL intensity of the Cu-Cy nanoparticles with the increase in size. Additionally, the possibility of better crystallinity could be playing some role for the higher PL intensity with the increase in the size of Cu-Cy nanoparticles.

Basically, we did not see much noticeable changes in the peak positions. The emissions are from the d-d transitions of the copper ions in two sites [15], which are mainly suppressed by the crystal field strength and mainly determined by the electronegativity and the chemical bond length. However, it is possible that the crystal field strength could be disturbed by the particle size as that particles become smaller, the changes of the ligand ions at the near surfaces would affect the chemical bonding [40]. Nonetheless, this effect is not so obvious as the quantum size confinement on the emission of excitons or band-to-band transitions in semiconductor quantum dots [42].

**Figure 3:**
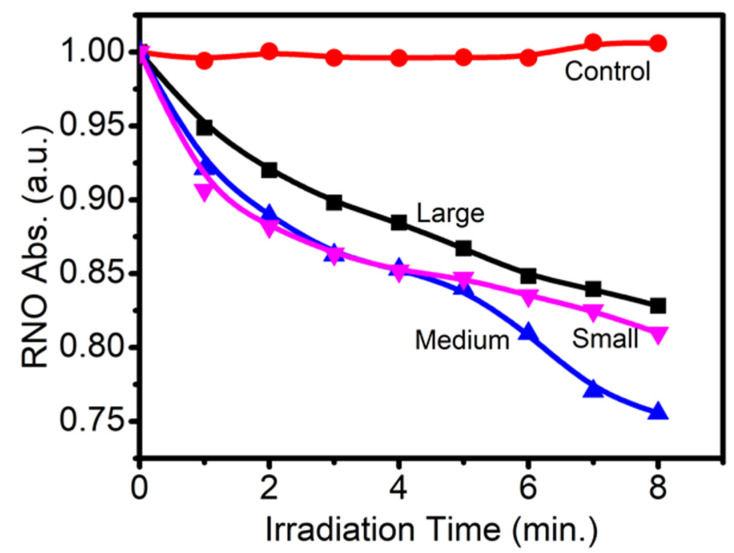
Reactive oxygen species (ROS) produced by the large, medium, and small-sized Cu-Cy nanoparticles upon X-ray irradiation (90 kVp) using the *p*-nitrosodimethylaniline and imidazole (RNO-ID) method.

We also measured the ROS produced by different sized Cu-Cy nanoparticles upon X-ray irradiation by using the RNO-ID assay and the results are presented in Figure 3. Interestingly, the 300 nm Cu-Cy nanoparticles produced the lowest amount of ROS. The PL intensity and ROS generation of a photosensitizer are two competing de-excitation pathways. Consequently, there is an inevitable trade-off between them. Owing to the fact that highly efficient ROS-producing photosensitizers are often weak fluorophores and vice versa [43,44], large-sized nanoparticles should have the highest PL intensity and lowest ROS production. As expected, the large-sized Cu-Cy nanoparticles showed the highest PL intensity (Figure 2) and produced the lowest amount of ROS (Figure 3). Furthermore, the amount of ROS generated by nanoparticles is related to particle shape, size, surface charge, solubility, aggregation status, and chemical structure [45]. Since smaller nanoparticles have larger surface areas and the size of nanoparticles regulates the number of reactive sites on the surface of nanoparticles [45], this could be another reason for producing a lower amount of ROS by the large-sized Cu-Cy as compared to small or middle-sized Cu-Cy nanoparticles. However, the relations among the particle size, the PL intensity, and the ROS production are complicated. It is not easy to illustrate them in a simple way.

### 3.2. Longitudinal Model of Tumor Size Response

**Figure 4:**
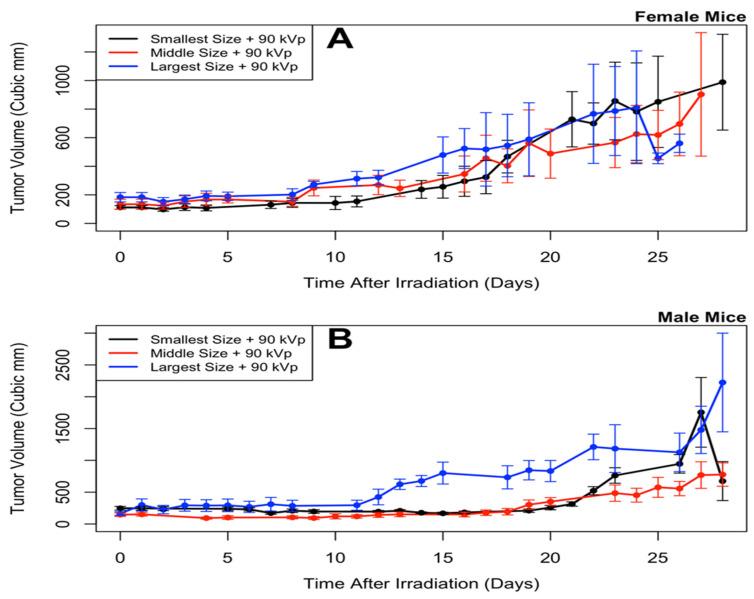
Tumor size versus time, for different nanoparticle sizes, in female (**A**) and male (**B**) mice. Mean and standard error of the mean are plotted; points without error bars came when only one mouse was left alive in a treatment group. Statistical analyses of these results indicate that smaller sizes produce the smallest tumors.

**Figure 5:**
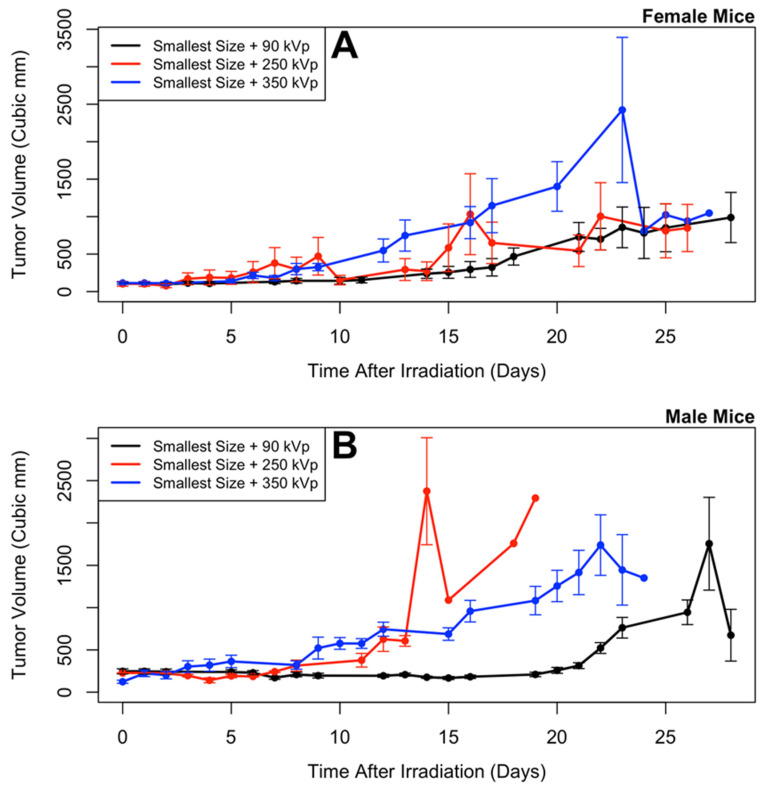
Tumor size versus time, for different radiation energies, in female (**A**) and male (**B**) mice. Mean and standard error of the mean are plotted; points without error bars came when only one mouse was left alive in a treatment group. Statistical analyses of these results indicate that lower energies produce the smallest tumors.

Tumor size as a function of time is shown in Figure 4 and Figure 5, and the analysis results are given in Table 1 and Table 2. The results of the longitudinal analysis (Table 1) show that the following experimental variables had a statistically significant effect on tumor size: log(time), log(time)^2^, sex, age at irradiation, tumor volume at irradiation, radiation energy, and nanoparticle size. The significance of log(time) and log(time)^2^ suggests that the tumor volume behaved exponentially with time. The statistically significant difference (*p* = 0.0323) seen between males and females (and clearly visible in the Figure 4 and Figure 5) may be because breast cancer was used for this experiment, and breast cancer can behave differently in males than in females [46]. Radiation response is known to change with age [47]. Tumor volume at time of irradiation is an initial value for the experiment; a larger initial value would mean a larger value at later time points compared to starting from a smaller initial value.

**Figure 6:**
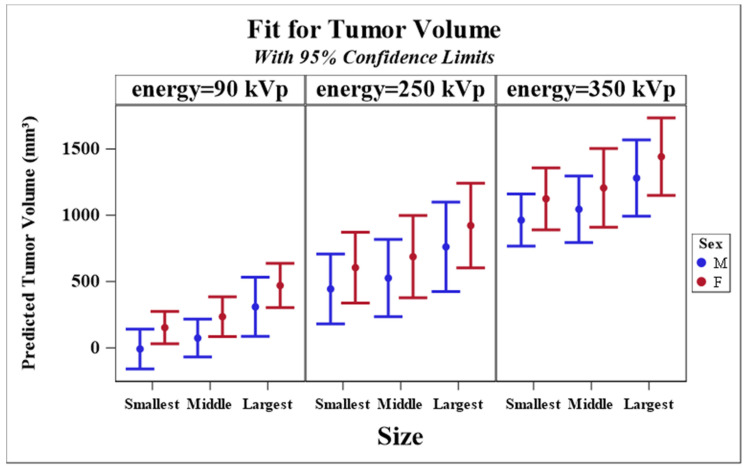
The predicted values of tumor size by different radiation energies, nanoparticle sizes, sex, computed at log(time) = 0, age at irradiation = 0, and tumor volume at irradiation = 0. Estimates and 95% confidence limits of the predicted values are plotted.

Table 2 shows the level of statistical significance (in the longitudinal analysis from Table 1) between each value of nanoparticle size and between each value of radiation energy. Each value of radiation energy is different by a statistically significant amount, with the lowest energy (90 kVp) having the lowest tumors volumes and the highest energy (350 kVp) having the highest tumor volumes. With nanoparticle size, the smallest size resulted in the lowest tumor volumes, and the largest size resulted in the highest tumor volumes; however, after correction for multiple testing the only statistically significant difference was between the largest and smallest sizes. Similar results are observed in Figure 6.

### 3.3. Missing Data Model

The missing data model, summarized in Table 3, shows that log(time)^2^ had a significant negative effect and suggests that mice were less likely to be observed as time increased. Tumor volume at previous day and 250 kVp radiation energy also had significant negative effects, implying that mice with larger tumor volume at previous day and 250 kVp radiation energy were more likely to die and thus less likely to be observed in the present day.

## 4. Discussion

The results of tumor size suggest that lower radiation energies and smaller sizes of nanoparticles (average size 40 nm) may result in significantly lower tumor volumes, although the middle size of nanoparticles was not different by a statistically significant amount from the smallest size or the largest size. However, smaller nanoparticles fluoresced less after irradiation than the other sizes, and the middle-sized nanoparticles (average size 100 nm) were found to produce the highest amount of reactive oxygen species upon X-ray irradiation.

The conditions for the (90 kVp) treatment are the same as those described in our previous work [20]. The overall behavior of the tumor volume is the same—similar size for a time period, then the same increase. However, the period of time is shorter in these mice. The results of Table 1 show that many variables affect the tumor size.

Basic radiation theory may partially explain the effect of nanoparticle size and radiation energy seen in the tumor size experiment. Regarding the effect of radiation energy, higher radiation energies tend to impart more energy per interaction (see, for example, Tables A-4 in [24]). Thus, for a given dose (energy/mass), there will be fewer interactions overall. The effect of Cu-Cy (producing ROS after an interaction with radiation) may scale linearly with the number of interactions. Thus, fewer interactions could mean less ROS for a given amount of imparted energy.

Regarding the effect of nanoparticle size in the tumor experiment, we used the same amount of Cu-Cy nanoparticles for all sizes. Consequently, there would have been fewer larger nanoparticles. For the larger nanoparticles (average size 300 nm), it is possible that the ROS released in the interior of the nanoparticle may not have enough energy to reach the outside of the nanoparticle. A similar argument, based on simulations, was made about Auger electrons in gold nanoparticles [25]. Perhaps this explains the reduction in ROS output of larger nanoparticles compared to medium size (average 100 nm) nanoparticles. If that is the case, the amount of ROS reaching cells could be less, per mass of Cu-Cy nanoparticles.

Based on our previous study on the size effect in gold nanoparticles [28], where sizes in the range of roughly 20 nm outperform smaller nanoparticles, it is possible that the trend of smaller sizes doing better would end at a certain point. This is very similar to our recent observations on skin cancer treatment [49], where Cu-Cy nanoparticles of an average size of 96 nm have been used. It was found that these Cu-Cy nanoparticle-based X-PDT exhibited a remarkable antitumor effect towards SCC. However, B16F10 melanoma was resistant to these Cu-Cy nanoparticle based X-PDT, both in vitro and in vivo [49], whereas the 40 nm Cu-Cy nanoparticles are very effective in inhibiting melanoma under X-ray stimulation [50]. This could be due to the fact that the 40 nm Cu-Cy nanoparticles have a larger surface area, thereby producing more ROS and the cellular uptake is higher for the 40 nm nanoparticles [18]. Accordingly, we believe that both the ROS production as well as the cellular uptake determines the treatment outcomes.

This work demonstrates that lower radiation energies are more effective with Cu-Cy nanoparticles, compared to higher radiation energies. Lower radiation energies have higher interaction probabilities. In fact, the 90 kVp X-ray energy (90 kilovolts peak) may actually catch a significant portion of the uptick in interaction probabilities around to the k-edge of copper (8.98 keV). Using the National Institute of Standards and Technology(NIST) database, and approximating 90 kVp as 30 keV, 250 kVp (with external Thoraeus-1 shielding) as 125 keV and 350 kVp (with external Thoraeus-1 shielding) as 175 keV, we estimate that the 90 kVp interactions are roughly 40 times higher than 250 kVp (due to a 40 times larger total cross section) and roughly 60 times higher than 350 kVp. It is important to note that this result does not rule out the possibility that the combination of Cu-Cy nanoparticles with higher radiation energies could still enhance radiation therapy, just perhaps to a lesser extent than with lower radiation energies.

This study represents a first step towards translating Cu-Cy nanoparticles from a demonstrated proof of concept toward clinically relevant applications. Future steps may include evaluations of intravenous delivery, biodistribution assays (similar to [32]), fractionated radiation treatments, higher and lower radiation energies, and smaller nanoparticle sizes. Cu-Cy nanoparticles have the potential to become a viable option for enhancing radiation therapy in cancer patients, after more work to establish the effect of experimentally important variables.

## 5. Conclusions

In summary, for the first time, the effect of nanoparticle size and the energy of X-rays on Cu-Cy nanoparticle-based X-ray-PDT are investigated. We found that the effects of the particle size on their performance is complex, while for energy, 90 kVp is more effective than 250 kVp or 350 kVp radiation energies for PDT activation. These observations provide useful information to optimize the nanoparticles as well as the excitation energies for photodynamic effects on cancer destruction.

## Figures and Tables

**Table 1:** Results of longitudinal analysis on tumor zize. Tumor size was analyzed as a function of log(time), log(time)^2^, sex, age at irradiation, tumor volume at irradiation, radiation energy and nanoparticle size, with sex = M, energy = 90 kVp, and size = smallest being the reference level. A positive (negative) estimate indicates that a larger (smaller) value of the parameter is associated with a larger tumor size than the reference level. A *p*-value < 0.05 is considered statistically significant. Results show that every variable had a statistically significant effect on the tumor size, although the middle and smallest sizes were not different by a statistically significant amount.

Parameter Estimates for Response Model				
**Parameter**		**Estimate**	**Standard Error**	***p* Value**
**Intercept**		−0.8984	0.1490	<0.0001
**Log(time)**		−1.5387	0.1143	<0.0001
**Log(time)^2^**		0.6422	0.0446	<0.0001
**Sex**	F	0.3136	0.1465	0.0323
**Sex**	M	0	-	-
**Age at Irradiation**		−0.2552	0.0545	<0.0001
**Tumor Volume at Irradiation**		0.3751	0.0814	<0.0001
**Energy**	250 kVp	0.8810	0.2854	0.0020
**Energy**	350 kVp	1.8928	0.2220	<0.0001
**Energy**	90 kVp	0	-	-
**Size**	Middle	0.1599	0.1678	0.3407
**Size**	Largest	0.6183	0.1864	0.0009
**Size**	Smallest	0	-	-

**Table 2:** Comparisons of energy and size. Estimate is equal to the estimate of the parameter in the first column (from Table 1) minus the estimate of the parameter in the second column (from Table 1); thus a positive (negative) estimate indicates that the parameter in the first column is associated with a larger (smaller) tumor size. *p* values were adjusted for multiple testing using the Tukey–Kramer method [48]. An adjusted *p* < 0.05 is considered statistically significant.

Size Comparisons					
**Size 1**	**Size 2**	**Estimate**	**Standard Error**	***p* Value**	**Adjusted *p* Value**
Middle	Largest	−0.4584	0.2063	0.0263	0.0675
Middle	Smallest	0.1599	0.1678	0.3407	0.6068
Largest	Smallest	0.6183	0.1864	0.0009	0.0026
**Energy Comparisons**					
**Energy 1**	**Energy 2**	**Estimate**	**Standard Error**	***p* Value**	**Adjusted *p* Value**
250 kVp	350 kVp	−1.0118	0.3534	0.0042	0.0117
250 kVp	90 kVp	0.8810	0.2854	0.0020	0.0057
350 kVp	90 kVp	1.8928	0.2220	<0.0001	<0.0001

**Table 3:** Results of missing data model. A positive (negative) estimate indicates that a larger (smaller) value of the parameter is associated with a higher probability of mice being euthanized. A *p* value < 0.05 is considered statistically significant.

Parameter Estimates for Missing Data Model				
**Parameter**		**Estimate**	**Standard Error**	***p* Value**
**Intercept**		3.6207	3.9875	0.3639
**Log(time)**		5.4121	3.1964	0.0904
**Log(time)^2^**		−1.6934	0.6434	0.0085
**Tumor Volume at Previous Day**		−0.6733	0.1315	<0.0001
**Sex**	F	−0.6169	0.3835	0.1077
**Sex**	M	0	-	-
**Age at Irradiation**		−0.1016	0.2423	0.6750
**Tumor Volume at Irradiation**		−0.0135	0.2244	0.9520
**Energy**	250 kVp	−1.4274	0.6902	0.0386
**Energy**	350 kVp	−0.5345	0.6811	0.4326
**Energy**	90 kVp	0	-	-
**Size**	Middle	−0.0749	0.6110	0.9025
**Size**	Largest	−0.4060	0.5793	0.4834
**Size**	Smallest	0	-	-

## Supplementary Materials

The following are available online at https://www.mdpi.com/2079-4991/10/6/1087/s1, Table S1: List of mice (identified by ear tag number), treatment variables (nanoparticle size and radiation energy) and reason for eventual euthanization.
