# Supplementary material for: Effects of Nanoparticle Size and Radiation Energy on Copper-Cysteamine Nanoparticles for X-ray Induced Photodynamic Therapy
Source: Nanomaterials (Basel). 2020 Jun 1;10(6):1087. doi: 10.3390/nano10061087 (PMC7353381; doi:10.3390/nano10061087)
Supplement: Supplementary file 1 [file nanomaterials-10-01087-s001.pdf]

Supplementary Materials:

Effects of Nanoparticle Size and Radiation Energy on Copper-Cysteamine Nanoparticles for X-ray Induced Photodynamic Therapy

Table S1. List of mice (identified by ear tag number), treatment variables (nanoparticle size and radiation energy) and reason for eventual euthanization.

Mouse Number	Reason for Euthanasia	Radiation Energy	Nanoparticle Size
359	Large tumor	90 kVp	Smallest
361	One month	90 kVp	Middle
379	Large tumor	90 kVp	Smallest
380	Large tumor	90 kVp	Smallest
381	Large tumor	90 kVp	Smallest
382	Necrotic	90 kVp	Smallest
383	Necrotic	90 kVp	Smallest
402	One month	90 kVp	Middle
405	One month	90 kVp	Middle
407	Necrotic	90 kVp	Middle
409	Necrotic	250 kVp	Smallest
414	Large tumor	250 kVp	Smallest
416	Necrotic	250 kVp	Smallest
417	One month	250 kVp	Smallest
419	Large tumor	250 kVp	Smallest
421	Large tumor	250 kVp	Smallest
422	Large tumor	250 kVp	Smallest
425	Large tumor	250 kVp	Smallest
428	Large tumor	90 kVp	Largest
429	One month	90 kVp	Largest
430	Large tumor	90 kVp	Largest
431	Large tumor	90 kVp	Largest
437	Large tumor	350 kVp	Smallest
438	Large tumor	350 kVp	Smallest
439	Necrotic	350 kVp	Smallest
448	Large tumor	90 kVp	Smallest
449	Large tumor	90 kVp	Smallest
450	Large tumor	90 kVp	Middle
451	One month	90 kVp	Middle
452	One month	90 kVp	Middle
453	One month	90 kVp	Largest
454	Large tumor	350 kVp	Smallest
455	Necrotic	350 kVp	Smallest
456	Necrotic	350 kVp	Smallest
457	Necrotic	350 kVp	Smallest
465	Large tumor	250 kVp	Smallest
466	Necrotic	250 kVp	Smallest

469	Necrotic	90 kVp	Largest
470	One month	90 kVp	Largest
471	One month	90 kVp	Largest
472	Necrotic	90 kVp	Middle
473	One month	90 kVp	Middle
474	One month	90 kVp	Smallest
476	Necrotic	90 kVp	Smallest
477	One month	90 kVp	Middle
478	One month	90 kVp	Largest
480	Necrotic	350 kVp	Smallest
481	Large tumor	350 kVp	Smallest
484	Large tumor	350 kVp	Smallest
486	Necrotic	350 kVp	Smallest
487	Large tumor	350 kVp	Smallest
488	Large tumor	90 kVp	Largest
489	Large tumor	90 kVp	Largest
500	Necrotic	90 kVp	Largest
607	One month	90 kVp	Middle
608	One month	90 kVp	Middle
609	One month	90 kVp	Middle
611	One month	90 kVp	Smallest
612	One month	90 kVp	Smallest
613	One month	90 kVp	Smallest
